# Effects of Dietary Food Components on Cognitive Functions in Older Adults

**DOI:** 10.3390/nu13082804

**Published:** 2021-08-16

**Authors:** Hitoshi Ozawa, Taiki Miyazawa, Teruo Miyazawa

**Affiliations:** New Industry Creation Hatchery Center (NICHe), Tohoku University, Sendai 980-8579, Japan; hitoshi.ozawa.b8@tohoku.ac.jp (H.O.); taiki.miyazawa.b3@tohoku.ac.jp (T.M.)

**Keywords:** aging, brain lipids, cognitive function, dietary food components, phospholipids, polyphenols, older adults, oxidative stress, reactive oxygen species, vitamins

## Abstract

Population aging has recently been an important issue as the number of elderly people is growing worldwide every year, and the extension of social security costs is financially costly. The increase in the number of elderly people with cognitive decline is a serious problem related to the aging of populations. Therefore, it is necessary to consider not only physical care but also cognitive patterns in the future care of older adults. Since food contains a variety of bioactive substances, dietary patterns may help improve age-related cognitive decline. However, the relationship between cognitive function and individual food components remains ambiguous as no clear efficacy or mechanism has been confirmed. Against this background, this review summarizes previous reports on the biological process of cognitive decline in the elderly and the relationship between individual compounds in foods and cognitive function, as well as the role of individual components of food in cognitive function, in the following order: lipids, carotenoids, vitamins, phenolic compounds, amino acids, peptides, and proteins. Based on the research presented in this review, a proper diet that preserves cognitive function has the potential to improve age-related cognitive decline, Alzheimer’s disease, and Parkinson’s disease. Hopefully, this review will help to trigger the development of new foods and technologies that improve aging and cognitive functions and extend the healthy life span.

## 1. Introduction

All countries worldwide are on the track of a super aging society in the current era. The global population aged over 60 years has doubled in the last 30 years and is expected to double again by 2050 [1]. Japan has the highest ratio of elderly people over 65 years of age globally, as well as the highest life expectancy and healthy life expectancy as reported by the Ministry of Health, Labour and Welfare (MHLW). However, the aging of the population leads to high financial costs due to the extension of social security costs and stagnant economies, and the number of elderly people with dementia increases at the same time. The World Health Organization reported approximately 50 million people with dementia worldwide, with almost 10 million new cases occurring each year [2]. The importance of the issue of cognitive aging was reported [3], and many studies examined predictors of nursing home placement in the elderly [4]. The loss of cognitive function takes away independence and can significantly affect family members [5]. Therefore, it is necessary to consider not only physical care but also cognitive patterns in the future care of older adults. Preserving cognitive function maintains independence in older adults, resulting in major social and financial benefits. The relationship between dietary patterns, dementia, and Parkinson’s disease (PD) has been widely debated to date [6,7]. Daily dietary consumption can be an important factor in maintaining cognitive function. Nutritional research aimed at maintaining healthy cognitive and metabolic functions often focused on individual food components. Foods and nutrients were identified that may prevent cognitive decline, including fruits, vegetables, fish, monounsaturated fats, polyunsaturated fats, and antioxidants. The association between dietary patterns, cognitive function, and metabolic syndrome was reported in older adults [8]. For example, the Mediterranean diet is known to reduce the risk of cardiovascular disease, cancer, and diabetes, and was recently associated with better cognitive function in the elderly population [9]. Furthermore, cognitive dysfunction associated with metabolic syndrome was reported [10], and the effects of metabolic syndrome on cognitive performance in adults during exercise were also reported [11]. Therefore, the development of functional foods for disease prevention is of great importance in the transition of disease properties. However, the relationship between cognitive function and individual food components remains ambiguous, as no clear efficacy or mechanism has been confirmed. Against this background, this review summarizes previous reports on the biological process of cognitive decline in the elderly and the relationship between individual compounds in foods and cognitive function, and describes the role of individual food components in cognitive function.

## 2. Method

To carry out the literature search, Google Scholar, Pubmed and Web of Science were employed. The search was based on key words such as aging, Alzheimer’s disease, amino acid, antioxidants, brain, CL-HPLC, carotenoid, cholesterol, cognitive decline, cognitive function, curcumin, dementia, dietary, elderly people, fatty acid, food, free radical theory, lipid peroxidation, luminol, older adult, oxidation, oxidative stress, Parkinson’s disease, peptide, phenolic compound, phospholipid, plasmalogen, polyphenol, protein, reactive nitrogen species, reactive oxygen species and vitamin.

## 3. Biological Reactions Associated with Aging

### 3.1. Reactive Oxygen Species, Reactive Nitrogen Species and Free Radical Theory

The involvement of free radicals in cognitive decline and aging has been widely recognized and discussed. Free radicals are universally produced during aerobic cell metabolism. In the body, antioxidant systems work against the free radicals generated to maintain homeostasis. However, due to aging or disease, when the antioxidant system of the body does not work properly or is overexposed to reactive oxygen species (ROS), the major components of the cells, such as lipids [12], proteins [13,14], and DNA [15,16,17], are oxidatively damaged. Oxidative damage to cells and tissues caused by ROS is considered to be reduced to some extent by the body’s enzymatic and non-enzymatic antioxidant defense systems, but the “free radical theory” was proposed to suggest that oxidative damage may outweigh this reduction [18,19].

**Figure 1:**
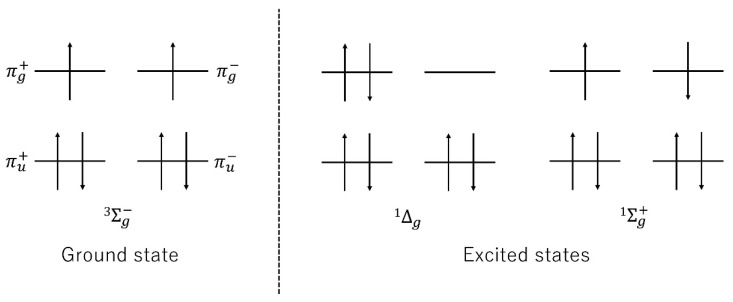
Electron configuration of the π orbitals of the oxygen molecule.

In general, ROS include singlet oxygen (specifically the ^1^$\Delta_{g}$ state), superoxide anions, hydrogen peroxide, and hydroxyl radicals. The ground state of the oxygen molecule is the ^3^$\Sigma_{g}^{-}$ state, and there exists a ^1^$\Delta_{g}$ state and a ^1^$\Sigma_{g}^{+}$ state in the excited state (Figure 1) because the orbital energies of each π orbital (two bonding orbitals and two antibonding orbitals) degenerate in homonuclear diatomic molecules. It is considered that only the ^1^$\Delta_{g}$ state is related to the reaction with other compounds because the energy of the ^1^$\Sigma_{g}^{+}$ state is higher than that of the ^1^$\Delta_{g}$ state and transition to the ^1^$\Delta_{g}$ state occurs. Meanwhile, the ground state of oxygen molecules can easily become super oxide anions because the ground state of the oxygen molecule is biradical. Super oxide anions are monoradicals because of the lone pair electrons. Superoxide anions are produced in various biological systems. Singlet molecular oxygen and hydroxyl radicals, which have high reactivity, are produced from superoxide anions. Hydrogen peroxide is produced by the two-electron reduction of triplet molecular oxygen. More chemically unstable ROS tend to be more reactive.

ROS cause cumulative oxidative stress to neural tissue in the brain, resulting in significant impairment of cognitive function, such as Alzheimer’s disease (AD), PD, and aging. Antioxidants are expected to be effective in the reduction in oxidative damage caused by free radicals, the removal of ROS or its precursors, and the binding to metal ions that is required to catalyze ROS production [20]. In particular, the supply of antioxidant food components to the body has attracted attention for its potential to inhibit the progression and onset of AD, PD, and aging, and the importance of daily meals has been discussed.

Moreover, it is known that nitric oxide plays an important role as a cell-signaling molecule, anti-infective agent and antioxidant [21]. Nitric oxide and its derivatives, such as nitrosothiols, peroxynitrite, nitronium cation and nitrogen dioxide, are called reactive nitrogen species (RNS) [21,22,23]. Nitric oxide is produced within cells by the actions of a group of enzymes called nitric oxide synthases. It is able to form other reactive intermediates, which could have an effect on protein function and on the function of the entire organism. These reactive intermediates can trigger nitrosative damage on biomolecules and may lead to age-related diseases due to the structural alteration of proteins, the inhibition of enzymatic activity, and interferences in regulatory function [22]. Furthermore, it is suggested that the modulatory effects exerted by ROS and RNS on ion channel proteins might have a relevant role in neuronal cell survival or death but more work is required to establish the possible involvement of ion channels and of their modulation by ROS and RNS as important mechanisms of the aging process [23].

### 3.2. Lipid Peroxidation

**Figure 2:**
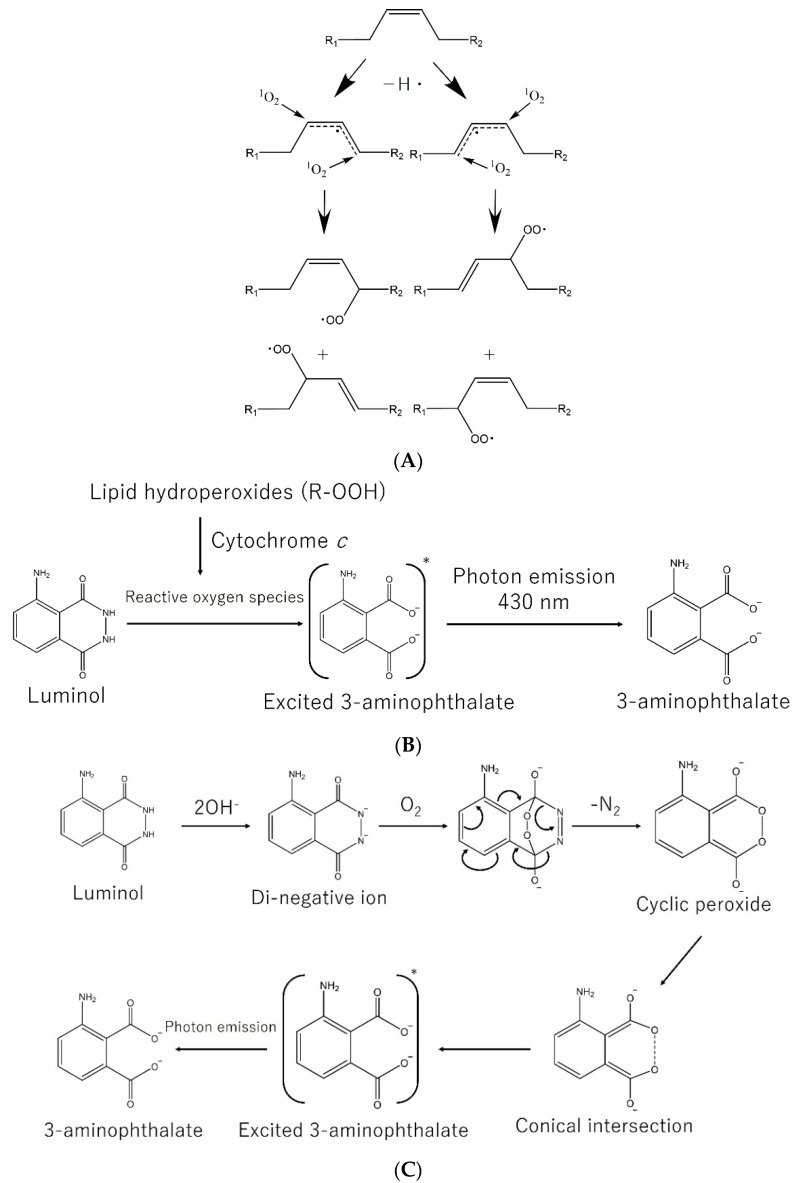
Free radical peroxidation of lipid by singlet oxygen (**A**) and chemiluminescence reaction of luminol with cytochrome *c* and lipid hydroperoxide (**B**) and proposed reaction scheme of luminol reaction (**C**). * indicates the excited state. Modified from [36,41,42] with permission from Elsevier and the American Chemical Society.

Research on lipid peroxidation in food deterioration, lipid nutrition, and age-related diseases has gained significant attention in terms of improving societal health and longevity [24]. In 1988, Miyazawa discovered that phospholipid hydroperoxides, which are the first oxidized form of membrane phospholipids, are present in the human body [25]. In the main components of foods and organisms (lipids, proteins, and carbohydrates), lipids are the most reactive oxygen molecules. The resonance of the π bond stabilizes radicals generated by hydrogen atom transfer in lipids, and lipid hydroperoxides are produced by the reaction of lipid radicals and ROS. The free radical peroxidation of lipids by singlet oxygen is shown in Figure 2A. Elucidating the formation of lipid peroxides due to oxidative stress is important not only for food quality, atherosclerosis, and cancer, but also for the suppression of aging. AD is the most common type of cognitive impairment, and in recent years, many studies have reported oxidative changes in the blood of AD patients [26,27,28,29,30,31]. The blood of patients with AD has been reported to have a higher concentration of phospholipid hydroperoxide-rich erythrocytes (aged erythrocytes) than that of healthy individuals. Aged erythrocytes are believed to be one of the causes of the onset and progression of AD because they can lead to insufficient oxygen supply and the deterioration of blood rheology. It is believed that high concentrations of amyloid β accumulate in the brain of patients with AD and cause oxidative stress in neurons, some of which are released into the bloodstream and adhere to erythrocyte cell membranes. Amyloid β attached to the erythrocyte membrane promotes lipid peroxidation in the cell membranes [32]. Amyloid β40 and amyloid β42 attached to human erythrocytes were quantified; their adhesion was reported to increase with age, and there is a positive correlation between amyloid β attached to human erythrocytes and lipid hydroperoxides in erythrocyte membranes [33]. The appearance of oxidized lipoproteins such as low-density lipoprotein and high-density lipoprotein due to aging is also related to pathological conditions such as hyperlipidemia, atherosclerosis, myocardial infarction, and stroke [34,35].

Miyazawa et al. developed a chemiluminescence high performance liquid chromatography (CL-HPLC) system, which enabled the detection of phosphatidylcholine hydroperoxide (PCOOH) in biological samples [25,36,37]. This method uses luminol (5-amino-2,3-dihydro-1,4-phthalazinedione) [38,39] and detects photon emission during the oxidation of luminol by the reaction of lipid hydroperoxides and cytochrome *c* (Figure 2B) [36,40]. The proposed reaction scheme for the luminol reaction is shown in Figure 2C [41,42]. Di-negative ions and cyclic peroxide structures exist as intermediates in the luminol reaction. The intersystem crossing of 3-aminophthalate from the lowest triplet state to the lowest singlet excited state has been proposed in an earlier study [41], but it has been proposed, in theoretical calculations, that the conical intersection between the ground state and the singlet excited state, on the potential energy surface of the reaction process from the cyclic peroxide structure to 3-aminophthalate, controls luminol chemiluminescence [42]. Several reports have confirmed the reduction in lipid peroxides in human organisms when foods are ingested using the CL-HPLC method. Miyazawa et al. reported that daily intake of chlorella tablets (8 g chlorella/day/person) for more than 1 month suppressed the appearance of aged erythrocytes in the blood of senior Japanese participants [43]. Burdeos et al. reported that 3 weeks of ingestion of rice bran oil rich in tocotrienol (5 or 10 mg of tocotrienol/day) reduced triglyceride and phospholipid hydroperoxide levels in the blood and liver of F344 rats [44]. However, the detailed mechanism by which such foods reduce phospholipid peroxidation in vivo is still unclear. In recent years, new analytical methods for lipid peroxidation in vivo involving LC-MS/MS were established one after another, and integrated elucidation of the mechanism of lipid peroxidation using such analytical methods is expected [24]. Recently, ferroptosis, which is a newly identified regulated form of cell death correlated with the etiopathogenesis of PD and AD, was considered to play a major role in neurodegenerative disease [45]. Ferroptosis is regulated mainly via iron homeostasis, glutathione metabolism and lipid peroxidation. Hence, the importance of the association between lipid peroxidation and cognitive impairment should increase in future studies.

## 4. Role of Functional Food Compounds in Cognitive Performance

### 4.1. Major Phospholipids

Phospholipids have a hydrophobic tail containing two fatty acids and a hydrophilic head containing a polar group. Glycerophospholipids (GPLs) are key components of cell membranes. They are the major source of long-chain polyunsaturated fatty acids (PUFA) and the reservoir of signaling molecules. Glycerophospholipids in cell membranes mainly include phosphatidylcholine (PC), phosphatidylethanolamine (PE), phosphatidylserine (PS), and phosphatidylinositol (PI) [46]. They can be derived from de novo synthesis or resynthesis. For example, in enterocytes, dietary GPL is hydrolyzed to free fatty acids and lysophospholipids [47,48,49]. PC and PS were investigated for their roles in brain development, with some positive effects on cognitive enhancement in preclinical models [50,51] and mixed results in clinical studies [52,53]. Sphingomyelin (SM) is a sphingophospholipid that is classified as either a phospholipid or a sphingolipid. SM is particularly rich in the myelin sheath of the central nervous system [54,55], and due to its role in myelin integrity [56] and axonal maturation [57], SM was implicated in several different cognitive disorders [58]. The effects of aging on the content, composition, and synthesis of SM in the central nervous system were reported [59].

**Figure 3:**
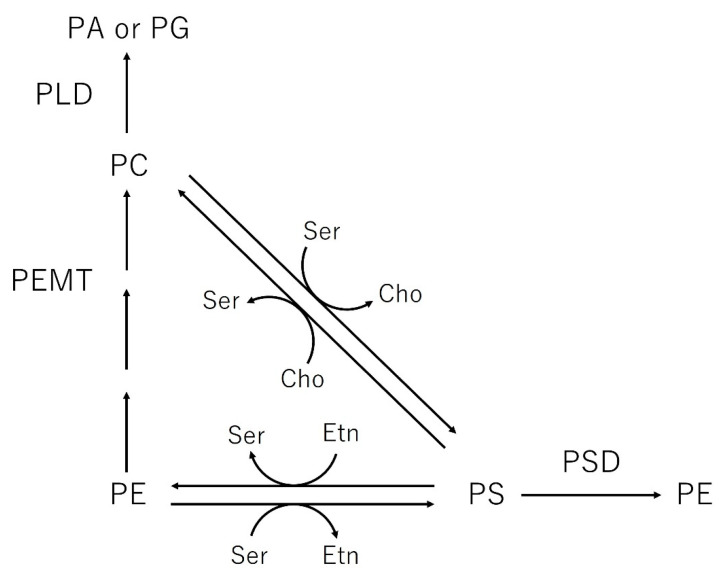
Alternative pathways for phospholipid synthesis in the brain (PA is phosphatidic acid). Ser: serine, Cho: choline, Etn: ethanolamine. Modified from [60] with permission from Elsevier.

Brain vulnerability increases rapidly with age and its deterioration has been elucidated in a variety of ways. One feature of the aging process is the modification of the properties of the brain membrane [60]. It has been observed that the composition of lipid fatty acids in the brain membrane and, consequently, the fluidity of the membrane change with increasing age [61]. Phospholipids are a key factor in their properties because cell membranes are mainly composed of phospholipids. Studies carried out in rats up to 18 months of age indicated that the synthesis of phospholipids such as PC and PE in the brain decreased during aging [62,63]. Furthermore, it has been clarified that the main phospholipid of nerve cell membranes, which plays an important role in supporting neural functions, is PS [64]. PS has been shown to play an important role in the release of acetylcholine, dopamine, and noradrenaline [65]. Many animal studies have provided evidence that PS plays an important role in age-related changes in brain function in aging [66,67]. Oral administration of exogenous PS to aged rats promoted the formation of synapses, dendrites, and surface receptors of nerve cells in different parts of the brain [66,68]. Exogenous phosphatidylserine facilitates the normalization of age-related decreases in Na^+^/K^+^-ATPase activity in brain cells [69], optimizes the secretion and reception of several neurotransmitters [70,71,72], and plays an important role in the control of signal messengers [66]. Alternative pathways for phospholipid synthesis were proposed in different areas of the brain during aging [60]. These pathways include base exchange enzymes, which are calcium-dependent, energy-independent, and calcium-stimulated enzymatic pathways, PE synthesis through phosphatidylserine decarboxylase activity, PC synthesis through the transfer of methyl groups to endogenous PE via phosphatidylethanolamine N-methyl transferase activity, and the synthesis of phosphatidylglycerol (PG) through phospholipase D (PLD) (Figure 3).

Since animal products (eggs, meat, etc.) are the major dietary sources of polar lipids, the relationship between dietary polar lipids and the development of cognitive function has been discussed [73]. In particular, the role of dietary phospholipids in cognitive processes throughout life is considered to be of great importance [74]. Regarding the impact of phospholipids on cognitive development, PC and PS are the most widely studied [74]. The distribution of phospholipids in the brain and the main dietary sources are shown in Table 1.

Dietary phospholipids were shown to have several health benefits, including improved cognitive function over a lifetime [76,77]. In humans, cognition encompasses and evaluates all biological processes, including attention, learning, memory, reasoning, judgment, decision-making, problem solving, and understanding. In rodents, cognition is primarily assessed by measuring learning, memory, and attention [78].

Many studies have confirmed that supplementation with phospholipids from dairy products can improve cognitive function, which is disrupted by stress and aging (Table 2). The effects of a diet rich in phospholipids on cognitive deficits related to aging have been explored in preclinical studies. One study used a diet supplemented with complex milk lipid concentrate (CMLc) that was rich in phospholipids [79], and another used oral administration of PS isolated from krill or soy [80]. Guan et al. treated 24-month-old rats with gelatin containing CMLc for 4 months [79]. As a result, old rats that orally ingested CMLc showed reduced errors in the probe test in the Morris water maze test. They also reported that compared to the control rats, CMLc-supplemented aged rats showed improved vascular density, dopamine output, and neuroplasticity in the brain regions involved in memory. Lee et al. investigated the effect of oral administration of krill-derived PS (20 or 50 mg/kg body weight/day) and soy-derived PS (50 mg/kg body weight/day) for one week on improving age-related learning and memory impairment in 6-week-old and 12-month-old rats [80]. The escape latency in the Morris water maze was significantly improved in rats fed with krill-derived PS than those fed with soybean-derived PS. Furthermore, in both groups, the age-associated loss of cholinergic immunoreactivity and muscarinic acetylcholine receptor type 1 (mAChR-M1) and choline transporter (CHT) mRNA expression in the hippocampus was significantly alleviated.

Several human studies on the effects of PS intake on cognitive function were also reported. Previous research showed that PS intake could compensate for cognitive deficits related to old age and AD in humans [81,82]. Cenacchi et al. evaluated the therapeutic efficacy and safety of oral PS versus placebo (300 mg/day for 6 months) in 494 elderly patients with cognitive impairment in a double-blind study [81]. The PS group showed significant improvement in both behavioral and cognitive aspects when compared to the placebo group. Engel et al. conducted a double-blind crossover study of phosphatidylserine (300 mg/day for 8 weeks) versus placebo in 33 patients with mild primary degenerative dementia (55–75 years old, Mini-Mental State Examination (MMSE) score 15–27) [82]. The results indicated that the PS group showed significant improvement in both behavioral and cognitive aspects when compared to the placebo group. As explained in this section, since phospholipids are essential for brain function, it is clear that their long-term intake from food has a significant impact on cognitive function.

### 4.2. Plasmalogens

**Figure 4:**
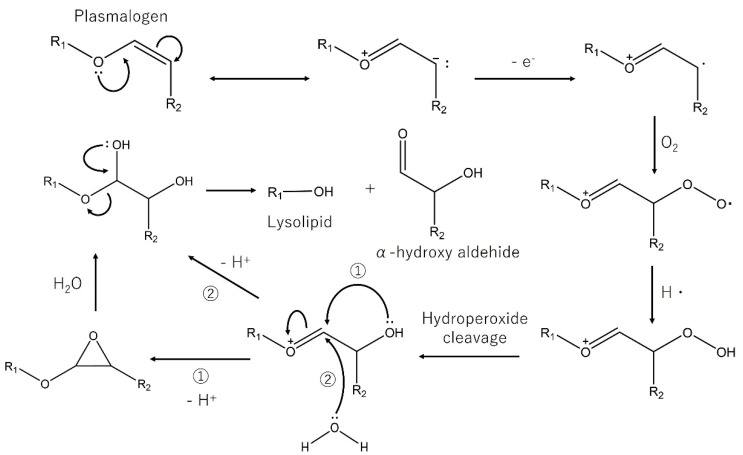
Proposed oxidative mechanism of plasmalogen. Modified from [88] with permission from The American Society for Biochemistry and Molecular Biology.

Plasmalogen is an alkenyl acyl formed lipid, which has a vinyl ether bond. Half of the brain is lipid, and plasmalogen accounts for approximately 8% of the brain [86]. Plasmalogen was also reported to prevent neuronal cell death by scavenging singlet oxygen and superoxide anions with its alkenyl (vinyl ether) linkages [87]. The proposed oxidative mechanism of plasmalogen is shown in Figure 4 [88].

Plasmalogen levels were reported to specifically decrease in the postmortem brains of patients with AD [89,90,91]. Plasmalogen was found to exist in *Halocynthia roretzi*, and eicosapentaenoic acid (EPA) and docosahexaenoic acid (DHA) are the main compounds [92,93,94]. Furthermore, ethanolamine plasmalogen, which is present at high levels in the brain, is believed to be involved in neuronal protection. The various food items (food resources derived from cattle, pigs, chickens, and marine products) examined showed a wide range of ethanolamine plasmalogen contents from 5 to 549 μmol/100 g wet weight [94]. Marine invertebrates, blue mussels, and ascidians had a high ethanolamine plasmalogen content (over 200 μmol/100 g wet weight). The profiling of the molecular species showed that the predominant fatty acids of ethanolamine plasmalogen species were 20:5 (EPA) and 22:6 (DHA) at the *sn*-2 position of the glycerol moiety in marine foodstuffs, whereas the major ethanolamine plasmalogen species in land foodstuffs were 20:4. Following quantitative analysis by multiple reaction monitoring, the viscera of the ascidian contained the highest levels of 18:0/20:5-ethanolamine plasmalogen and 18:0/22:6-ethanolamine plasmalogen (86 and 68 μmol/100 g wet weight, respectively). To evaluate the neuronal antiapoptotic effect of these ethanolamine plasmalogen species, human neuroblastoma SH-SY5Y cells were treated with PE, purified from the ascidian viscera, under serum starvation conditions. Extrinsic PE from ascidian viscera showed stronger suppression of cell death induced by serum starvation than from bovine brain glycerophospholipids. The glycerophospholipid from ascidian viscera strongly suppressed the activation of caspase 3. Therefore, ethanolamine plasmalogens, especially those containing EPA and DHA, from marine foodstuffs, are potentially useful as a therapeutic dietary supplement to prevent neurodegenerative diseases such as AD. Moreover, it was found that plasmalogen, which originates in sea squirt, is an anti-apoptosis factor in nerve cells and the administration of plasmalogen improves long and short-term dementia [33]. One article also reported a significant improvement in cognitive function and clinical symptoms such as AD and PD with the elevation of blood plasmalogen levels [95]. Although these findings suggest that plasmalogen has important physiological functions, its absorption and metabolism are not fully understood, mainly due to the lack of plasmalogen resources [96,97].

### 4.3. ω3. Fatty Acids

Long-chain ω3 fatty acids, such as EPA and DHA, are important for brain function and mental health [98,99,100], and several studies showed an association between ω3 fatty acids and cognitive performance (Table 3).

Therefore, the consumption of fish as food can contribute to the improvement of cognitive function [107,108,109,110,111]. A large study conducted under the National Health and Nutrition Examination Survey (NHANES) in 2011–2014 reported an association between dietary intake of ω3 and ω6 fatty acids and cognitive function in the elderly. A total of 2496 participants aged 60 years and older were included in the study [112,113,114]. The NHANES study reported the following results: in the fully adjusted model, the odds ratios and 95% confidence intervals for the Consortium to Establish a Registry for Alzheimer’s Disease (CERAD) test score, Animal Fluency test score, and DANTES Subject Standardized Test (DSST) score were 0.58 (0.38–0.88), 0.68 (0.47–0.99), and 0.59 (0.37–0.99), respectively, for the upper to lower quartiles of ω3 fatty acid intake. Meanwhile, the odds ratios and 95% confidence intervals for the CERAD test score, Animal Fluency test score, and DSST test score were 0.48 (0.31–0.75), 0.60 (0.40–0.92), and 0.590 (0.34–0.75) for the upper to lower quartiles of ω6 fatty acid intake, respectively. The association between the ω6:ω3 ratio and cognitive performance was not statistically significant in the three tests. In the dose–response relationship analysis, L-shaped associations were apparent for ω3 and ω6 fatty acid intake in relation to the CERAD test score, animal fluency test score, and DSST test score. Therefore, dietary ω3 and ω6 fatty acid intake may be inversely associated with low cognitive performance. Previous reports on the effects of dietary ω3 and ω6 fatty acids on cognitive decline yielded contradictory results, making it difficult to conclude its usefulness at this time. Further studies will clarify the relationship between ω3 and ω6 fatty acid intake and cognitive function in the future.

### 4.4. Cholesterols

The relationship between cholesterol and cognitive function has not yet been definitively determined; however, there have been several studies. Earlier results suggested a link between high midlife cholesterol levels and increased risk of dementia [115,116,117,118], while later studies did not find an association between midlife cholesterol and subsequent dementia [119,120,121,122]. Furthermore, studies on late-life cholesterol levels contradicted some studies reporting that low late-life cholesterol was associated with an increased risk of dementia, although others did not find an association [123,124,125]. In a recent study, the relationship between cholesterol levels and cognitive function depended on homocysteine levels, suggesting an interactive role between cholesterol and homocysteine in cognitive function in the elderly population [126]. Moreover, it has also been reported that the consumption of arachidonic acid and cholesterol through dietary fat intake may be associated with an increased risk of PD [127]. Furthermore, niacin, which increases high-density protein [128], could play a key role in the improvement of PD because one study has shown an association between high-density lipoprotein cholesterol variability and a risk of developing PD [129].

### 4.5. Carotenoids

Carotenoids have all trans-type conjugated double bonds and are considered to be antioxidants and important compounds in the longevity of primate species [130,131]. Therefore, the carotenoid supply of food reduces oxygen stress and contributes to the aging epidemic disease by increasing its concentration in the body. The rate of electron transfer from the carotenoid radical anion to oxygen was previously studied [132]. The hypothesis that carotenoids play a preventive role in cognitive impairment is suggested by their ability to trap peroxyl radicals and their singlet oxygen-quenching properties, allowing them to prevent lipid peroxidation [132,133]. Epidemiological studies and clinical trials on cognitive impairment and plasma carotenoids are mainly concerned with β-carotene, which is a major carotenoid [134,135,136,137,138,139,140]. On the other hand, several studies showed that the antioxidant activity of other carotenoids could be more effective than β-carotene activity [141,142]. Specifically, the role of lutein in cognitive function throughout life is important [143], and a possible role of lutein in cognitive function in the elderly was reported [144]. In this study, 49 women (age, 60–80 years) were randomly assigned to receive DHA (800 mg/d; n = 14), lutein (12 mg/d; n = 11), a combination of DHA and lutein (n = 14), or placebo (n = 10). Participants underwent cognitive tests measuring verbal fluency, memory, processing speed, and accuracy and provided self-reports of mood at randomization and at the end of the trial. After supplementation, verbal fluency scores improved significantly in the combined treatment group, and individuals in this group also showed a trend toward more efficient learning. Measures of mental processing speed, accuracy, and mood were not affected by supplementation. Therefore, this study suggests that DHA and lutein improve cognitive function in older adults. Among erythrocyte antioxidants, xanthophyll lutein in AD was lower than in healthy participants, and lutein tended to decrease as the level of AD increased [35]. It was also reported that amyloid β induces oxidative injury to erythrocytes by binding to them, causing phospholipid peroxidation and diminishing xanthophylls and lutein [145]. A similar trend to that of lutein was reported with astaxanthin (a carotenoid that was reported to exhibit stronger antioxidant activity in vitro than lutein) [146]. In addition, the possibility of lutein supply through the ingestion of *Chlorella pyrenoidosa* was suggested because it includes lutein in abundance [147,148], and it has been reported that its dietary intake increases lutein concentration in blood [149].

### 4.6. Vitamins

Vitamin and antioxidant enzymes play a fundamental role in protecting the organism from oxidative stress, which is associated with longevity [150]. The major natural antioxidant vitamins, most of which are derived from natural sources by dietary intake, are vitamin C, vitamin E, and vitamin A [150]. The link between vitamin C status and cognitive performance in cognitively intact and impaired individuals (including AD and dementia) was evaluated [151]. Some studies showed a significantly lower vitamin C blood concentration in cognitively impaired individuals than in healthy individuals [152,153,154]. Furthermore, vitamin E, particularly α-tocopherol, is the major chain-breaking antioxidant in plasma [155] and cell membranes [156]. The function of vitamin E as a regulatory redox interaction was reported [157]. A link between vitamin E and aging, dementia, and AD was suggested because of its antioxidant function [158]. Furthermore, the importance of vitamin A in brain function, behavior, and learning was argued [159]. In light of the recent attention given to the effects of micronutrients on human cognition, it is surprising that vitamin A received relatively little attention compared to other vitamins [160,161]. Vitamin A and its derivatives, which are essential for human health, modulate several physiological processes through their interactions with the nuclear retinoic acid receptors (RARs) and retinoid X receptors (RXRs) [162]. High levels of vitamins A and E appear to be important in guaranteeing extreme longevity [150]. Plasma levels of ascorbic acid, uric acid, α-tocopherol, retinol, carotenoids, total thiol groups, and the activity of plasma superoxide dismutase (SOD) and glutathione peroxidase (GPX), as well as the activity of red blood cell (RBC) SOD, were measured. Participants were divided into the following four groups according to age: 32 healthy centenarians, 17 elderly participants aged 80–99 years, 34 elderly participants aged 60–79 years, and 24 adults aged less than 60 years. Considering only the non-centenarians, consistent behavior in the antioxidant pattern, with a decrease in non-enzymatic antioxidants and an increase in enzymatic antioxidant activity relative to age, was observed. Remarkably, centenarians were characterized as having the highest levels of retinol and α-tocopherol, whereas the activities of plasma and RBC SOD, which increased with age, decreased in centenarians. From these results, healthy centenarians showed a particular profile in which high levels of retinol and α-tocopherol seemed to be important to guarantee their extreme longevity. However, a later study reported different antioxidant profiles of vitamin A and E in Italian centenarians [163]. Furthermore, the roles of vitamins B, D, and K in aging adults were also reported [164,165,166,167,168]. For example, apolipoprotein E (APOE) ε4 is a genetic predisposing factor that modulates the effect of vitamin B-12 on cognitive function [164]. Neuropsychological tests, including the MMSE for global cognition, were administered at the baseline assessment to 539 Chinese adults (≥55 years). MMSE was repeated at a median of 18 months (n = 376) and 38 months (n = 247) after baseline. The interaction of vitamin B-12 and APOE ε4 with cognitive function was examined using a linear mixed effects model for MMSE and a multiple linear regression model for neuropsychological test scores. As a result, APOE ε4 was associated with a lower MMSE score. Vitamin B-12 was positively related to the MMSE score, and this association was much stronger in APOE ε4 carriers than in APOE ε4 noncarriers. Significant interactions between natural log-transformed vitamin B-12 and APOE ε4 were also found for the digit span backward longest sequence, and for immediate recall in the Rey auditory verbal learning test. Better performance in these two tests was associated with vitamin B-12 in APOE ε4 carriers but not in APOE ε4 noncarriers. Therefore, the association between vitamin B-12 and cognitive function was moderated by APOE ε4 status. In addition, the effect of low vitamin D on cognition and the relationship between vitamin D and PD were reported [167,169].

### 4.7. Phenolic Compounds

The use of potent antioxidant nutritional substances, such as (poly)phenols, was proposed in the study of age-related cognitive disorders [170,171,172]. The effects of the consumption of flavonoids and other polyphenols on cognitive performance were discussed, and the findings of previous human experimental and epidemiological studies imply that the consumption of polyphenols has the potential to benefit cognition both acutely and chronically [173]. Recent data from randomized placebo-controlled trials suggest that (poly)phenols may also modulate neurological disorders, cerebral hypoperfusion, and neuroinflammation while simultaneously improving memory, learning, and cognitive performance in older adults (Table 4) [170,174,175,176,177,178,179,180,181,182].

However, other studies reported nonsignificant effects or even unwanted effects of (poly)phenol-rich supplementation on certain cognitive functions, specifically executive functioning, working memory, and verbal memory [183,184], or the response of cerebral blood flow, and are thus controversial [185]. Among various polyphenolic compounds, curcumin is considered a promising therapeutic agent for altering the cognitive symptoms of AD, and several preclinical studies were conducted to verify its efficacy [186,187]. One of the main reasons for this is that it was reported to bind to amyloid β plaques, decrease neurotoxicity, and initiate their degradation. However, only a limited number of clinical studies examined the effects of curcumin on human cognitive functioning. Some studies did not report cognitive-enhancing effects of curcumin [188,189], while other studies suggested a beneficial effect of curcumin on cognition [190,191]. Some studies suggest that curcumin is responsible for mechanisms that are protective against cognitive decline [188,191]. The results concerning the reduction in amyloid β are ambiguous since most of the peripheral measurements, such as plasma, serum, and CSF (Chalder Fatigue Scale) levels, did not detect significant changes in amyloid β or tau levels between curcumin and placebo [188,192]; however, neuroimaging supports that curcumin reduces amyloid β deposits in the brain [192]. Most orally administered curcumin undergoes glucuronidation and sulfate conjugation during Phase 1 and Phase 2 metabolism in the liver and small intestine before entering the bloodstream [193,194]. Since the physiological and antioxidant effects of curcumin are known to decrease after undergoing metabolism (conjugation), it will be necessary to evaluate curcumin and cognitive function while taking metabolism into account in the future [195,196].

### 4.8. Amino acids, Peptides, and Proteins

As introduced in the previous chapter, amyloid β was suggested to play an important role in the development and progression of AD. The elucidation of the pathological mechanisms of amyloid β42 in AD attracted interest in relation to the discovery of new drugs [197]. Therefore, the role of peptides in cognitive function is important. In addition to long-form peptides, small oligopeptides are also very important compounds in cognitive function. For example, it was hypothesized that lactotripeptide ingestion increases cerebral blood flow and cognitive function [198]. Age-related decreases in cerebral blood flow velocity increase the risk of cerebrovascular disease. Bioactive peptides derived from milk proteins, such as lactotripeptide, were shown to inhibit angiotensin-converting enzyme (ACE) activity and increase vasodilator production. ACE is a central component of the renin-angiotensin system, which controls blood pressure by regulating the volume of fluid in the body and converts the hormone angiotensin I to the active vasoconstrictor angiotensin II. Valine-proline-proline (VPP) and isoleucine-proline-proline (IPP) exist in lactotripeptides. Furthermore, Met-Lys-Pro (MKP), a casein-derived ACE inhibitory peptide with the potential to cross the blood–brain barrier, was also reported to attenuate cognitive decline in a mouse model of AD [199]. Milk peptide (casein hydrolysate (CH)-3), which contains MKP and adult male ddY mice, was used in this study. An animal model of AD was induced by intracerebroventricular (ICV) injection of amyloid β1-42. CH-3 (250 mg/kg/day) or MKP (0.5 mg/kg/day) was administered orally every day from 2 days before ICV injection. Three weeks after ICV injection, cognitive function was evaluated using the Morris water maze test. Daily administration of CH-3 markedly attenuated amyloid β 1-42-induced cognitive decline. The effects of MKP on human cognition were also investigated [200]. Furthermore, imidazole-containing amino acids and dipeptides are known as antioxidants in general [201,202]; carnosine in the histidine metabolism pathway has been reported to decrease in AD plasma [203]. Moreover, glutathione is also known as an antioxidant for the cellular detoxification of ROS in the brain [45,204]. Recently, Ozawa et al. developed a method for the comprehensive analysis of dipeptides and showed that the profiles of dipeptides in the body were greatly altered in diseases such as cancer [205,206]. Clarifying the dynamics of dipeptides in food and the body will be important to elucidate the mechanism of cognitive decline. Meanwhile, protein/amino acid intake is a growing area of research related to the prevention of AD and related dementia [207], and consumption is directly related to a number of disease-related risk factors such as low muscle mass, poor sleep, stress, depression, and anxiety. As a result, the role of protein/amino acid intake in terms of affecting modifiable risk factors for cognitive decline has provided a robust area for scientific exploration. However, this research is still speculative and specific mechanisms have to be proven.

### 4.9. Others

In other studies, dietary boron, brain function, and cognitive performance were reported [208]. Penland evaluated the relationship between brain electrophysiological responses to boron intake (about 0.25 mg boron/2000 kcal/day and approximately 3.25 mg boron/2000 kcal/day) and cognitive performance in healthy elderly men and women (65 people in total). They reported that performance was significantly lower in manual dexterity, eye-hand coordination, attention, perception, encoding, and short-term memory and long-term memory tests when boron intake was low as opposed to when boron intake was high. The effect of dietary nitrate on cognitive function in the elderly has been the subject of some debate, although no definite effect has been reported [209,210]. In these reports, dietary nitrate supplementation reduced resting blood pressure and improved O_2_ uptake kinetics during treadmill walking in healthy older adults, but did not improve walking or cognitive performance. Therefore, nitrate supplementation was shown to improve exercise performance and cardiovascular response, but its benefits on cognitive performance are unclear. Moreover, there is a discussion about whether caffeine is a cognitive enhancer [211,212]. Over 26 years, up to 2000, global coffee consumption increased by 67.9%. Therefore, elucidation of the relationship between cognitive function and caffeine will be of increasing interest [213].

## 5. Conclusions

In this review, the effects of various food components on cognitive function are presented based on previous reports. Dementia is an important problem in a modern aging society. Based on the research presented in this review, a proper diet that preserves cognitive function could improve age-related cognitive decline, AD, and PD. As mentioned in this review, there are many types of functional components in food. However, there is no direct evidence that any food component improves cognitive function, and the effect of food components on cognitive function remains unclear. An understanding of foods composed of various components will be necessary for future research on cognitive functions. Reactive oxidative species are deeply associated with aging, and biomolecules are oxidatively damaged according to the “free radical theory”. Hence, the function of food compounds as antioxidants is expected to improve age-related disorders. Food compounds that have these functions are included in daily meals. Therefore, maintaining dietary habits is important to maintaining health in old age. New quantitative methods have been developed for compounds related to cognitive functions (lipid peroxidation, dipeptides, etc.) that have been difficult to analyze in the past. These methods of analyzing foods and the human body will contribute to the discovery of new functions of foods to improve dementia in an aging society. Hopefully, this review will help to trigger the development of new foods and technologies that improve aging and cognitive functions and extend the healthy life span.

## Figures and Tables

**Table 1:** Brain distribution of phospholipids per mg of tissue and distribution of phospholipids in the main dietary sources expressed as percentage (%) of total phospholipids. Modified from [74] with permission from Elsevier. Furthermore, a small portion of data from [75] are included.

Samples	PE	PC	PS	PI	SM
Brain	55.2%	31.3%	8.0%	5.3%	n.d.
Milk	37.5%	26%	7.2%	6.3%	23%
Egg yolk	16.6%	76.9%	n.d.	n.d.	2.3%
Soybean	26.2%	44%	n.d.	14%	n.d.

PC: phosphatidylcholine, PE: phosphatidylethanolamine, PS: phosphatidylserine, PI: phosphatidylinositol, SM: Sphingomyelin.

**Table 2:** Supplementation with dairy phospholipids improves disrupted cognition in stress and aging.

Compounds	Species	Improved Cognitive Functions	References
Complex milk lipids	Rat	Spatial memory	[79]
Phosphatidylserine	Human	Learning and verbal memory	[81]
Phosphatidylserine	Human	Immediate nonverbal memory	[82]
1,2-dilinoleoyl-sn-glycero-3-phosphocholine	Mouse	Spatial memory	[83]
Milk fat globule membrane	Rat	Spatial memory	[84]
Bovine milk-derived phospholipid drink	Human	Attention switching	[85]

**Table 3:** Previous study on the relationship between ω3 fatty acids and cognitive function.

Study Samples	Results	References
Blood plasma of AD patients, patients of dementia and patients who are cognitively impaired but nondemented	Low levels of ω3 fatty acids in the plasma may be a risk factor for cognitive impairment and/or dementia.	[101]
Data from cross-sectional population-based study among subjects aged 45–70 years	Fatty fish and marine ω3 PUFA consumption was associated with a reduced risk and intake of cholesterol and saturated fat with an increased risk of impaired cognitive function in the middle-aged population.	[102]
Erythrocyte membrane of men and women (aged 63–74 years) from the Etude du Vieillissement Artériel (EVA) cohort	The inverse association between cognitive decline and the ratio of ω3 to ω6 fatty acid in erythrocyte membranes was confirmed.	[103]
Data derived from a cohort of men, aged 69–89 years, who were participants in the Zutphen Elderly Study	High linoleic acid intake was positively associated with cognitive impairment and high fish consumption inversely associated with cognitive impairment.	[104]
Plasma of adults aged 50–65 years	Promoting higher intakes of ω3 PUFA in the diet of hypertensive and dyslipidemic people may have substantial benefits in terms of reducing their risk of cognitive decline in the area of verbal fluency.	[105]
Plasma of men and women aged 50–70 years	Plasma ω3 PUFA proportions were associated with less decline in the speed-related cognitive domains over 3 years.	[106]

**Table 4:** Improvement of functions in older adults by daily intake of polyphenols.

Dietaries	Improved Functions	References
Cocoa with flavanol	Cognitive function, blood pressure control and metabolic profile	[174]
Blueberry	Episodic memory performance and cardiovascular disease	[175]
Greek mountain tea	Cognitive function	[176]
Dietary with resveratrol	Memory performance in association with improved glucose metabolism and increased hippocampal functional conectivity	[177]
Cocoa with flavanol	Regional cerebral perfusion	[178]
Blueberry	Brain perfusion and activation in brain areas associated with cognitive function	[179]
Fruit and 100% fruit juice	Cardiovascular disease, memory/cognition, obesity/diabetes and exercise performance	[180]
Soy with isoflavone	Cognitive function	[181]
Dietary with flavonol	Developing Alzheimer dementia	[182]
